# Are general and strategic measures of organizational context and leadership associated with knowledge and attitudes toward evidence-based practices in public behavioral health settings? A cross-sectional observational study

**DOI:** 10.1186/s13012-017-0593-9

**Published:** 2017-05-12

**Authors:** Byron J. Powell, David S. Mandell, Trevor R. Hadley, Ronnie M. Rubin, Arthur C. Evans, Matthew O. Hurford, Rinad S. Beidas

**Affiliations:** 1 Department of Health Policy and Management, Gillings School of Global Public Health, University of North Carolina at Chapel Hill, 1105C McGavran-Greenberg Hall, Campus Box 7411, Chapel Hill, NC 27599 USA; 2 Center for Mental Health Policy and Services Research, Department of Psychiatry, Perelman School of Medicine, University of Pennsylvania, Philadelphia, PA 19104 USA; 3 Community Behavioral Health, Philadelphia, PA 19107 USA; 4 Department of Behavioral Health and Intellectual disAbility Services, Philadelphia, PA 19107 USA; 5 American Psychological Association, Washington, DC USA; 6 Community Care Behavioral Health, Pittsburgh, PA USA

**Keywords:** Implementation research, Behavioral health, Organizational context, Attitudes toward evidence-based practice, Knowledge of evidence-based practices

## Abstract

**Background:**

Examining the role of modifiable barriers and facilitators is a necessary step toward developing effective implementation strategies. This study examines whether both general (organizational culture, organizational climate, and transformational leadership) and strategic (implementation climate and implementation leadership) organizational-level factors predict therapist-level determinants of implementation (knowledge of and attitudes toward evidence-based practices).

**Methods:**

Within the context of a system-wide effort to increase the use of evidence-based practices (EBPs) and recovery-oriented care, we conducted an observational, cross-sectional study of 19 child-serving agencies in the City of Philadelphia, including 23 sites, 130 therapists, 36 supervisors, and 22 executive administrators. Organizational variables included characteristics such as EBP initiative participation, program size, and proportion of independent contractor therapists; general factors such as organizational culture and climate (Organizational Social Context Measurement System) and transformational leadership (Multifactor Leadership Questionnaire); and strategic factors such as implementation climate (Implementation Climate Scale) and implementation leadership (Implementation Leadership Scale). Therapist-level variables included demographics, attitudes toward EBPs (Evidence-Based Practice Attitudes Scale), and knowledge of EBPs (Knowledge of Evidence-Based Services Questionnaire). We used linear mixed-effects regression models to estimate the associations between the predictor (organizational characteristics, general and strategic factors) and dependent (knowledge of and attitudes toward EBPs) variables.

**Results:**

Several variables were associated with therapists’ knowledge of EBPs. Clinicians in organizations with more proficient cultures or higher levels of transformational leadership (idealized influence) had greater knowledge of EBPs; conversely, clinicians in organizations with more resistant cultures, more functional organizational climates, and implementation climates characterized by higher levels of financial reward for EBPs had less knowledge of EBPs. A number of organizational factors were associated with the therapists’ attitudes toward EBPs. For example, more engaged organizational cultures, implementation climates characterized by higher levels of educational support, and more proactive implementation leadership were all associated with more positive attitudes toward EBPs.

**Conclusions:**

This study provides evidence for the importance of both general and strategic organizational determinants as predictors of knowledge of and attitudes toward EBPs. The findings highlight the need for longitudinal and mixed-methods studies that examine the influence of organizational factors on implementation.

## Background

There are a growing number of evidence-based practices (EBPs) for youth psychiatric disorders [1, 2], but they continue to be underutilized and poorly implemented in community settings [3–7]. To understand the relative lack of implementation success to date, a number of studies have examined the factors that facilitate or impede the adoption, implementation, and sustainment of EBPs (e.g., [8–15]). Implementation barriers have been summarized in a range of conceptual models and frameworks [16], including the Theoretical Domains Framework, the Consolidated Framework for Implementation Research [17], the Exploration, Preparation, Implementation, and Sustainment framework [18], and the Checklist for Identifying Determinants of Practice [19]. Nearly all of these models include multiple ecological levels and emphasize that effective implementation requires consideration of both provider and organizational levels. Successful implementation requires providers who view EBPs favorably and possess the knowledge and skill to deliver core components of interventions with fidelity, as well as organizational contexts that are sufficiently supportive of EBPs.

While individual- and provider-level factors are widely acknowledged to be critical to effective implementation, the literature is still in a nascent state in two respects. First, many conceptual models and frameworks do not specify relationships among individual and organizational constructs. This limits our ability to understand how these factors coalesce to influence implementation success or failure and, hence, how implementation strategies [20, 21] should be selected and sequenced to promote the implementation of EBPs.

Second, while some of the more general organizational constructs (e.g., organizational culture, organizational climate, leadership) have a relatively long history and have been well studied in a variety of fields [22, 23], many implementation-specific organizational constructs are emerging (e.g., implementation climate [24–26], implementation leadership [27], implementation citizenship [28]). Rather than assessing organizational characteristics and functioning more broadly, these constructs are theorized to be more proximal to implementation and to sharpen focus on the specific strategies that need to be enacted and the contexts that need to be created to ensure effective implementation [23]. The relationship between general and strategic (i.e., implementation-specific) organizational factors has yet to be established, and few studies have focused on whether they are uniquely predictive of individual-level factors (e.g., knowledge and attitudes) that are more proximal to implementation effectiveness [29, 30]. Developing a better understanding of the role and modifiability of these contextual factors is a priority for implementation research [31–33], as this knowledge could inform the careful development and testing of implementation strategies that could promote the implementation, sustainment, and scale-up of EBPs [20, 21, 34, 35]. The present study examined a set of general (organizational culture, organizational climate, transformational leadership) and strategic (implementation climate and implementation leadership) organizational factors and their association with clinician knowledge of and attitudes toward EBPs within the context of a large publicly funded behavioral health system in Philadelphia. We begin by defining each of these constructs and describing their relationship to implementation.

### Organizational culture and climate

Organizational culture and climate have a relatively long history in organizational research [23]. In the present study, we draw upon the work of Glisson and colleagues [36]. They define organizational culture as the shared views of norms and expectations within organizations [36]. Organizational climate refers to organizational members’ shared perception of the psychological impact of the work environment on their own well-being [36]. Organizational culture and climate have been associated with attitudes toward EBPs [37], provider turnover [38–40], quality of services [41], sustainment of new practices [39], and youth mental health outcomes [41, 42]. For further reference, Glisson and Williams [43] provide a thorough overview of work to develop more positive cultures and climates.

### Implementation climate

Implementation climate has been defined as employees’ shared perception that a specific innovation is expected, supported, and rewarded within their organization [44]. Strong implementation climates encourage the use of EBPs by (1) ensuring employees are adequately skilled in their use, (2) incenting the use of EBPs and eliminating any disincentives, and (3) removing barriers to EBP use [44]. Weiner and colleagues [24] have documented ways in which the conceptualization and measurement of implementation climate can advance the field. Perhaps most central to the present inquiry is that it may have greater predictive validity than more general constructs. The empirical evidence linking implementation climate to implementation effectiveness is primarily drawn from the information systems implementation literature [24]. However, two pragmatic measures for implementation climate have recently been developed by Ehrhart et al. [26] and Jacobs et al. [25], offering new opportunities for empirical work focusing on implementation climate.

### Transformational leadership

Effective leadership is essential for implementation success [17, 23] and can emerge (formally or informally) from any level of the organization [17]. Much work in this area has focused on the Full-Range Leadership model [45], which differentiates among transformational, transactional, and passive or laissez-faire leadership. Corrigan et al. [46] note that transformational leadership is characterized by helping team members transform their services to meet the ever-evolving needs of their patients and is achieved through charisma, inspiration, intellectual stimulation, and consideration of individual staff members’ interests. Transactional leaders attend to day-to-day tasks necessary for the program to operate, and they accomplish their goals by using goal-setting, feedback, self-monitoring, and reinforcement. Both transformational and transactional leadership styles have been shown to be effective, in contrast to laissez-faire leaders, who are characterized as aloof, uninvolved, and disinterested in the activities of the front-line workers [46]. However, transformational leadership is associated with more positive outcomes than is transactional leadership, including higher innovation climates and attitudes toward EBPs [47]. Aarons and colleagues [23, 48] provide informative reviews of empirical findings from applications of this model in a wide range of settings, including health, mental health, and child welfare.

### Implementation leadership

Aarons and colleagues [27] have developed the Implementation Leadership Scale (ILS), which measures specific behaviors that leaders exhibit to promote effective implementation. Implementation leadership is measured with four subscales: (1) proactive leadership, (2) knowledgeable leadership, (3) supportive leadership, and (4) perseverant leadership. A pilot study demonstrated that implementation leadership can be improved through an organizational implementation strategy called the Leadership and Organizational Change Intervention (LOCI) [49]; however, given that this measure was only recently developed, further research is required to establish its utility in predicting additional implementation determinants and outcomes.

### Knowledge of and attitudes toward evidence-based practices

Knowledge of EBPs is a precursor to implementation and is a key construct in some of the most commonly used implementation frameworks [17, 50]. For example, in child and adolescent mental health, it is necessary (although not sufficient) that therapists are knowledgeable about treatments that are indicated (or contraindicated) for common mental health problems such as anxiety and avoidance, depression and withdrawal, disruptive behavior, and attention and hyperactivity [51]. Therapists’ attitudes toward EBPs are also an important precursor to effective implementation. Aarons et al. [52] note that attitudes toward EBPs are important for two reasons. First, these attitudes may influence whether or not a therapist adopts a new practice. Second, if therapists do adopt a new practice, their attitudes may influence their decisions about the actual implementation and sustainment of the innovation. Measures have been developed to assess mental health therapists’ attitudes toward EBPs, including the 15-item Evidence-Based Practice Attitudes Scale (EBPAS). Studies using the EBPAS [53] have shown that organizational factors such as constructive organizational cultures and organizational support for EBPs are associated with more positive attitudes toward EBPs and that more positive attitudes toward EBPs are associated with provider adoption of EBPs [54–56]. However, Aarons and colleagues [52] underscore the need for more research that examines the association between organizational factors and attitudes toward EBPs.

### Study purpose and hypotheses

The purpose of this study was to (1) assess the relationship between general and strategic organizational-level determinants and therapist-level determinants of implementation and (2) to determine whether strategic organizational-level determinants predict individual-level determinants after controlling for general organizational-level determinants that are more firmly established in the implementation literature. We hypothesized that more positive general organizational determinants (i.e., organizational culture and climate, transformational leadership) would be associated with increased therapist knowledge of and more positive attitudes toward EBPs, because they are characterized by (1) more proficient organizational cultures in which therapists are expected to have up-to-date knowledge of best practices and they are less resistant to change [36]; (2) organizational climates that are more engaged, functional, and less stressful, which could facilitate the exploration of new programs and practices [36]; and (3) higher levels of transformational leadership, which has been shown to lead to more positive climates for innovation [47]. Moreover, we hypothesized that more positive strategic organizational contexts for implementation would be associated with higher levels of knowledge and more positive attitudes toward EBPs, as those contexts would be characterized by EBPs being expected, supported, and rewarded [24, 44] and by leaders who provide the concrete supports needed to facilitate the exploration (and ultimately the preparation, implementation, and sustainment) of EBPs [18, 27].

## Methods

### Setting

This study was conducted in partnership with Philadelphia’s Department of Behavioral Health and Intellectual disAbility Services (DBHIDS), a large, publicly funded urban system that pays for mental health and substance abuse services through a network of more than 250 providers. In partnership with Community Behavioral Health, a not-for-profit 501(c)(3) corporation contracted by the City of Philadelphia, they provide behavioral health coverage to more than 500,000 Medicaid-enrolled individuals [57]. Since 2007, DBHIDS has supported several large-scale initiatives to implement EBPs and has worked to develop a practice culture that embraces recovery and evidence-based care [58]. These initiatives have included efforts to implement cognitive therapy [59], trauma-focused cognitive behavioral therapy [60], prolonged exposure, dialectical behavior therapy, and, most recently, parent-child interaction therapy. In addition to providing robust training from treatment developers and other expert purveyors of these clinical practices, DBHIDS has attempted to address implementation challenges at the practitioner, organizational, and system levels through the use of a number of implementation strategies [58]. For example, DBHIDS utilized multilevel implementation strategies including forming an Evidence-Based Practice and Innovations Center (EPIC) to guide the overall vision of EBP implementation and address challenges related to communication, coordination, and institutional learning in the context of multiple EBP initiatives; establishing an EBP Coordinator role within EPIC; paying for clinicians’ lost billable hours during training; funding ongoing supervision and consultation; providing CEUs for EBP initiatives; providing enhanced reimbursement rates for some EBPs; giving public recognition to organizations implementing EBPs; ensuring that care managers and other DBHIDS staff members were well informed about EBPs; ensuring EBPs were billable under Medicaid; engaging a wide range of stakeholders to build buy-in and inform implementation; and sponsoring public events that focused on reducing stigma and raising awareness of behavioral health concerns. It is beyond the scope of this paper to describe DBHIDS’ transformation effort in great detail; however, it is described elsewhere. For example, Powell et al. [58] provide a broad overview of the effort by describing the aforementioned strategies (and more) and linking them to the policy ecology model of implementation [61]. Wiltsey Stirman et al. [59] and Beidas et al. [60] provide detailed descriptions of two individual initiatives focusing on cognitive therapy and trauma-focused cognitive behavioral therapy, respectively. The present study involved child- and youth-serving agencies and clinicians that were implementing a wide range of interventions and therapeutic techniques [56, 62].

### Agencies and participants

More than 100 community mental health agencies in Philadelphia provide outpatient services for children and youth (Cathy Bolton, PhD, written communication, January 3, 2013). Purposive sampling [63] was used to recruit the 29 largest child-serving agencies, which serve approximately 80% of children and youth receiving publicly funded mental health care. Of those 29 agencies, 18 (62%) agreed to participate and one additional agency that was involved with DBHIDS’ efforts to implement EBPs asked to participate, resulting in a final sample of 19 agencies (23 sites, 130 therapists, 36 supervisors, and 22 executive administrators). Due to their distinct leadership structures, locations, and staff, each site was treated as a distinct organization. The leader of each organization was invited to participate as the executive administrator. There were no exclusion criteria. Sixteen of the 23 participating organizations had participated in city-sponsored EBP initiatives, and those that did participate varied in terms of cumulative years of participation (see Table 2). We controlled for organizational participation in EBP initiatives in all analyses, which are described below. Additional studies focusing on the same sample of organizations have documented a myriad of barriers to implementing EBPs [15], including challenges related to turnover [40], a shifting workforce [64], and financial distress [65]. While all of these organizations operated within a broader system that was prioritizing and supporting the use of EBPs, variable participation in the initiatives and ample evidence that organizations were faced with challenges shared by most organizations in public service sectors minimize potential concerns about selection bias. However, we acknowledge that the baseline willingness to implement EBPs may be higher in this system given concerted efforts to prioritize and support EBP use.

### Procedure

The institutional review boards in the City of Philadelphia and at the University of Pennsylvania approved all study procedures. The executive administrator at each of the 23 organizations was asked to participate in the study. They completed their questionnaires using REDCap, a secure Web-based application that supports online data collection [66]. For supervisors and therapists, we scheduled a one-time, 2-h meeting at each organization, at which we provided lunch, obtained written informed consent, and completed data collection. Approximately 49% of therapists employed by the 23 organizations participated in the study. We collected data from March 1, 2013, through July 25, 2013. Participants received $50 for their participation.

### Measures

#### Predictor variables

##### Participant characteristics

Demographics were assessed using the Therapist Background Questionnaire [67].

##### Organizational characteristics

Three organizational characteristics were measured: number of therapists, proportion of staff that were independent contractors, and the total number of years each organization participated in each of the city-sponsored EBP initiatives (e.g., an organization that participated in the dialectical behavior therapy initiative for 2 years and the trauma-focused cognitive behavioral therapy initiative for 3 years would be coded as participating for 5 years in EBP initiatives). Data regarding the number of therapists and proportion of staff that were independent contractors was collected from organizational leaders, and data regarding EBP initiative participation was obtained from Community Behavioral Health and DBHIDS leadership. All three of these characteristics can be considered proxies for the amount of financial and human capital that may be available for EBP efforts. Financial difficulties [65] have led many organizations to employ an increasing number of therapists as independent contractors, and these therapists have been shown to have poorer attitudes toward and less knowledge of EBPs [64]. Participation in EBP initiatives gives organizations increased access to direct financial supports, including the provision of training and consultation related to a specific EBP. Controlling for these characteristics minimizes concerns about selection bias and informs the broader generalizability of this study to other contexts that may not have formal EBP initiatives underway.

##### Organizational social context (organizational culture and climate)

Organizational culture and climate were measured from the perspectives of therapists, supervisors, and executive administrators using the Organizational Social Context Measurement System (OSC) [36]. The OSC is a 105-item measure of organizational culture, climate, and work attitudes (the latter of which was not used for the current study). Organizational culture is measured using three second-order factors: proficiency, rigidity, and resistance. Proficient cultures are those in which employees prioritize the well-being of clients and are expected to be competent and have up-to-date knowledge. Rigid cultures are those in which employees have little discretion or flexibility in conducting their work. Resistant cultures are characterized by employees who show little interest in new ways of providing services and suppress any change effort. Organizational climate is also measured using three second-order factors, including engagement, functionality, and stress. Engaged climates are characterized by employee perceptions that they can personally accomplish worthwhile tasks, remain personally involved in their work, and maintain concern for their clients. Functional climates are characterized by employee perceptions that they receive the support that they need from coworkers and administrators to do a good job and that they have a clear understanding of their role and how they can be successful within the organization. In stressful climates, employees feel emotionally exhausted. Organizational culture and climate are measured with *T*-scores, in which a score of 50 represents the mean and the standard deviation is 10. These *T*-scores are based upon a normed sample of 100 community mental health clinics in the USA [36]. The OSC has strong psychometric properties [68], and the measurement model has been confirmed in two nationwide samples [36, 69].

##### Implementation climate

Implementation climate was measured from the perspective of therapists, supervisors, and executive administrators with the Implementation Climate Scale (ICS) [26]. The ICS is an 18-item scale that measures the climate for EBP implementation using six dimensions: focus on EBPs, educational support for EBPs, recognition for using EBPs, rewards for EBPs, selection of staff for EBPs, and selection of staff for openness. Each of the items is measured on a five-point Likert scale ranging from 0 (not at all) to 4 (very great extent), and each subscale represents the mean of the items within that dimension. There is strong support for the reliability and validity of the ICS, as well as the aggregation of the ICS and its subscales to the group level [26].

##### Transformational leadership

Transformational leadership was measured from the perspective of therapists who rated their direct supervisor using the Multifactor Leadership Questionnaire (MLQ 5X Short) [70]. The MLQ includes 45 items that measure transformational leadership, transactional leadership, and laissez-faire leadership. Given its association with innovation [47], we focused only on transformational leadership, which is measured using the 20 items and 3 subscales of the MLQ, including idealized influence (8 items), inspirational motivation (4 items), intellectual stimulation (4 items), and individual consideration (4 items). Each item is measured on a continuum from 0 (not at all) to 4 (to a very great extent), with each subscale representing the mean of the items within that dimension. The MLQ possesses strong psychometric properties and has been widely used in a variety of fields [70].

##### Implementation leadership

Implementation leadership was measured from the perspective of therapists who rated their direct supervisor with the Implementation Leadership Scale (ILS) [27]. The ILS is a 12-item scale that measures leader behaviors relevant to implementation of EBPs with four subscales, including proactive, knowledgeable, supportive, and perseverant. Each item is measured on a continuum from 0 (not at all) to 4 (very great extent), with each subscale representing the mean of the items within that dimension. The ILS has demonstrated excellent internal consistency reliability as well as convergent and discriminant validity [27].

#### Dependent variables

##### Knowledge of EBPs

Therapist knowledge of EBPs was measured using the Knowledge of Evidence-Based Services Questionnaire (KEBSQ) [51], a 40-item self-report instrument. Knowledge is measured on a continuum from 0 to 160, with higher scores indicating more knowledge of evidence-based services for youth. Psychometric data suggest temporal stability, discriminative validity, and sensitivity to training [51].

##### Attitudes toward EBPs

Therapist attitudes toward EBPs were assessed using the Evidence-Based Practice Attitudes Scale (EBPAS) [53], a 15-item self-report measure that assesses four dimensions: appeal of EBPs, likelihood of adopting EBPs given requirements to do so, openness to new practices, and divergence between EBPs and usual practice. Each item is measured using a scale ranging from 0 (not at all) to 4 (very great extent), and each of the aforementioned subscales represent the average of the items within that factor. Previous studies have demonstrated that the EBPAS has good internal consistency [54] and validity [71]. Aarons et al. [72] assessed the psychometric properties of the EBPAS in a study of 1089 mental health service providers from a nationwide sample drawn from 100 organizations in 26 states in the USA. The study provided support for the second-order factor structure of measure, provided further evidence of the reliability of its subscales and total scale, and yielded scale norms based upon a national sample [72].

### Data analysis

Organizational scores are constructed by aggregating individual responses within the organization; however, this is only meaningful if there is sufficient agreement within the organizational unit. We used mean within-group correlation statistics to determine agreement [73, 74]. The OSC, ICS, and ILS all had levels of agreement above the recommended 0.60 level [74, 75]; thus, individual participant responses within organizations were averaged.

Analyses were conducted using PROC MIXED in SAS statistical software, version 9.0 (SAS Institute, Inc.). We produced six linear mixed-effects regression models to determine the associations between the predictor variables (organizational characteristics, general and strategic organizational determinants) and dependent variables (knowledge of EBPs and attitudes toward EBPs). All models included random intercepts for organization to account for nesting of therapists within organizations and fixed effects for individual demographic and organizational factors. Demographic factors included age, sex, and years of clinical experience. Organizational characteristics included program size (number of therapists), ratio of independent contractor therapists, organizational culture (proficient, rigid, and resistant), organizational climate (engaged, functional, stressful), implementation climate (EBP focus, educational support, recognition, rewards, staff selection, openness), transformational leadership (idealized influence [attributed], idealized influence [behavior], inspirational motivation, intellectual stimulation, individualized consideration), and implementation leadership (proactive, knowledgeable, supportive, perseverant). The six dependent variables included knowledge of evidence-based services (KEBSQ) and attitudes toward EBPs (EBPAS; including scores for the four subscales [requirements, appeal, openness, and divergence] and the overall total).

## Results

### Descriptive statistics

Demographic information for the participating therapists is detailed in Table 1. Of the 22 executive administrators, 11 (50%) were male; they identified as Asian (2 [9%]), Hispanic/Latino (3 [14%]), African American (4 [18%]), white (12 [55%]), multiracial (2 [9%]), or missing ethnicity/race (2 [9%]). Of the 36 supervisors, 25 (69%) were female; they identified as African American (6 [17%]), white (20 [56%]), Hispanic/Latino (5 [14%]), other (1 [3%]), or missing ethnicity/race (4 [11%]). Descriptive statistics (medians, interquartile ranges) for the organizational variables (i.e., cumulative years participating in EBP initiatives, program size [number of therapists], proportion of staff employed as independent contractors, OSC subscales, ICS subscales, transformational leadership subscales, and ILS subscales) are shown in Table 2.

**Table 1:** Therapist demographics (*N* = 130)

Variable	Statistic
Race/ethnicity (*n* = 123 therapists)	
Asian	6 (5)
African American	27 (22)
White	67 (55)
Hispanic	13 (11)
Multiracial	5 (4)
Other	5 (4)
Educational background (*n* = 124 therapists)	
Bachelor’s degree	5 (4)
Master’s degree	107 (86)
Doctoral degree	12 (10)
Time at current organization, mean years (*n* = 124 therapists)	2 (1–4)
Sex (*n* = 129 therapists)	
Male	30 (23)
Female	99 (76)
Age, mean years (*n* = 122 therapists)	35 (29–47)
Clinical experience, mean years (*n* = 122 therapists)	5 (2–10)
Employment status (*n* = 119 therapists)	
Independent contractors	67 (56)
Salaried	52 (44)

*Note*. Data are presented as number (percentage) or mean (range)

**Table 2:** Descriptive statistics of organizational variables (*N* = 23)

Variable	Median (IQR)
Organizational characteristics	
Cumulative years participating in EBP initiatives	3.0 (0–5.0)
Program size (number of therapists)	9.5 (7.0–25.0)
Proportion of staff that are independent contractors	0.8 (0.4–0.9)
Organizational social context	
Proficient culture	55.6 (45.8–59.4)
Rigid culture	58.0 (53.0–63.2)
Resistant culture	64.2 (56.8–74.7)
Engaged climate	54.2 (48.8–58.7)
Functional climate	62.1 (55.3–72.2)
Stressful climate	55.5 (51.8–59.2)
Implementation climate	
Focus on EBPs	2.4 (1.8–2.9)
Educational support	1.6 (1.3–2.0)
Recognition	2.0 (1.7–2.6)
Rewards	0.4 (0.3–1.0)
Staff selection	2.3 (2.0–2.9)
Openness	2.9 (2.3–3.4)
Transformational leadership	
Idealized influence (attributed)	3.0 (2.6–3.3)
Idealized influence (behavior)	2.8 (2.3–3.3)
Inspirational motivation	3.0 (2.6–3.5)
Intellectual stimulation	2.6 (2.3–3.1)
Individualized consideration	2.8 (2.3–3.8)
Implementation leadership	
Proactive	2.1 (1.8–2.9)
Knowledgeable	2.9 (2.3–3.3)
Supportive	3.0 (2.7–3.4)
Perseverant	2.8 (2.4–3.3)

### Predictors of therapists’ knowledge of EBPs

Table 3 reports the results of the linear mixed-effects regression models of the association between organizational factors and knowledge of EBPs as measured by the KEBSQ. Five organizational variables were associated with knowledge of EBPs. General organizational factors that predicted knowledge included organizational culture, organizational climate, and transformational leadership. More proficient organizational cultures were associated with an increase in knowledge, whereas more resistant cultures were associated with a decrease in knowledge. More functional organizational climates were associated with a decrease in knowledge of EBPs. Transformational leadership (idealized influence [attributed]) was associated with increased knowledge of EBPs. The only strategic organizational factor that predicted knowledge was implementation climate, as higher ratings on the financial rewards for the EBP subscale of the Implementation Climate Scale were associated with decreased knowledge of EBPs.

**Table 3:** Variation in and factors associated with knowledge of (*n* = 127) and attitudes toward (*n* = 129) evidence-based practices

	KEBSQ 94.00 (89.50–101)	EBPAS Requirements 3.00 (2.00–3.67)	EBPAS Appeal 3.25 (2.67–3.75)	EBPAS Openness 3.00 (2.50–3.75)	EBPAS Divergence 1.25 (0.75–1.75)	EBPAS Total 3.00 (2.56–3.32)
Parameter estimates						
General organizational factors						
Organizational characteristics						
Cumulative years in initiatives	1.38	0.05	0.09	−0.06	−0.05	0.03
Program size	0.14	0.00	−0.00	−0.00	−0.00	0.00
Individual contractor ratio	−2.81	−0.33	−0.61	0.15	0.23	−0.26
Organizational social context						
Proficient culture	0.68*	−0.04	0.01	−0.04	−0.01	−0.02
Rigid culture	0.11	0.04	0.05*	−0.01	0.02	0.02
Resistant culture	−0.61*	−0.00	−0.03	0.02	0.01	−0.01
Engaged climate	−0.79	0.12	0.03	0.06	−0.05	0.07*
Functional climate	−1.10*	−0.00	−0.10*	0.04	0.05	−0.03
Stressful climate	−0.15	−0.10	−0.10*	0.00	0.00	−0.05
Transformational leadership						
Idealized influence (attributed)	10.23*	0.36	−0.16	−0.11	0.51	−0.10
Idealized influence (behavior)	−4.18	0.46	0.56	−0.00	1.06	−0.01
Inspirational motivation	−0.66	0.21	0.79	0.16	−0.88*	0.51
Intellectual stimulation	1.74	−0.40	0.03	0.19	−0.50	0.08
Individual consideration	−1.41	−1.33	−0.99*	−0.53	0.30	−0.79*
Strategic organizational factors						
Implementation climate						
Focus on EBPs	3.31	−0.45	0.12	0.18	0.02	−0.04
Educational support	3.18	1.95**	1.12*	0.16	−1.06*	1.07**
Recognition team	7.90	−0.38	−0.34	−0.10	−0.25	−0.14
Reward team	−8.51*	−0.32	−0.23	0.12	0.63*	−0.27
Staff selection	4.40	−1.62	−1.19	−0.25	0.50	−0.89
Selection openness	−3.77	0.60	1.05*	0.06	−0.38	0.52
Implementation leadership						
Proactive	−4.30	1.50	1.17*	0.57	0.04	0.80*
Knowledgeable	3.68	−0.17	0.38	−0.01	−0.59*	0.20
Supportive	−3.01	0.90	0.32	−0.29	−0.60	0.38
Perseverant	2.32	−2.48**	−1.94*	−0.46	0.91	−1.49**

*Note*. Values associated with the dependent variables in row 1 represent medians and interquartile ranges. These models also control for individual-level demographics, including gender, age, and years of experience

*<0.05; **<0.01

### Predictors of therapists’ attitudes toward evidence-based practices

Table 3 also reports the results of the linear mixed-effects regression models of the association between organizational factors and attitudes toward EBPs as measured by the EBPAS. General organizational factors that predicted attitudes toward EBPs included organizational culture, organizational climate, and transformational leadership. Organizational cultures characterized by higher rigidity ratings were associated with higher levels of appeal. Organizational climates that were more functional and stressful were associated with lower levels of appeal, and organizational climates that were more engaged were associated with higher total EBPAS scores. Transformational leadership also predicted attitudes toward EBPs. Inspirational motivation was associated with lower levels of perceived divergence. Individual consideration was associated with lower levels of appeal and total EBPAS scores.

Both strategic organizational factors (implementation climate and implementation leadership) were predictive of attitudes toward EBPs. Implementation climates characterized by higher levels of educational support were positively associated with higher ratings for requirements, appeal, and total attitudes toward EBPs, and negatively associated with divergence. Implementation climates that provided more monetary or tangible rewards for EBPs were associated with higher ratings for divergence. Selection for openness (implementation climate) was positively associated with appeal of EBPs. Proactive implementation leadership was associated with higher levels of appeal and total attitudes toward EBPs. Knowledgeable implementation leadership was associated with lower levels of perceived divergence. Finally, perseverant implementation leadership was associated with lower ratings of attitudes toward EBPs (requirements, appeal, and total attitudes toward EBPs).

## Discussion

This observational, cross-sectional study contributes to our understanding of how organizational factors are associated with clinician-level determinants of implementation in behavioral health [43, 48, 56, 76]. It confirms that both general and strategic organizational factors are associated with knowledge and attitudes toward EBPs. Indeed, at least one of the scales from each of the general (organizational culture, organizational climate, transformational leadership) and strategic (implementation climate, implementation leadership) constructs was associated with knowledge of or attitudes toward EBPs in the hypothesized direction.

### Associations between general organizational factors and knowledge and attitudes

More proficient organizational cultures were associated with higher knowledge of EBPs, which is consistent with the literature that suggests proficient cultures are characterized by having up-to-date knowledge of best practices [36, 37]. Conversely, resistant organizational cultures were associated with less knowledge of EBPs. This relationship is intuitive, as therapists working in resistant cultures are expected to show little interest in new ways of working and to suppress any change effort; thus, they are unlikely to seek and obtain new knowledge related to EBPs [36].

Organizational climates that were more engaged were associated with higher overall attitudes toward EBPs. Engaged climates are characterized by therapists’ perceptions that they are able to personally accomplish worthwhile things, remain personally involved in their work, and maintain concern for their clients [36]. Thus, organizational climates that are more engaged may provide fertile ground for therapists to orient toward personal growth and to nurture more positive attitudes toward EBPs. Organizational climates that were characterized as stressful were associated with lower ratings for appeal of EBPs. This is not surprising, as stressful climates are characterized by employees who feel exhausted, overworked, and unable to get necessary tasks completed [36]. It follows that they would find the prospect of learning about or using EBPs less appealing. More functional organizational climates were associated with lower knowledge of EBPs and lower appeal of EBPs. While this may seem counterintuitive, there does not seem to be as direct of a theoretical relationship between functional climates (i.e., those in which therapists receive the support and cooperation that they need to do a good job [36]) and attitudes toward EBPs as there is for other constructs, such as proficient cultures. Moreover, if therapists in more functional climates believe that they already have what they need to provide quality services and that the organization is functioning well without EBPs, they may not feel motivated to pursue EBPs.

Transformational leadership (idealized influence) was associated with higher levels of knowledge of EBPs and attitudes toward EBPs as inspirational motivation was associated with less perceived divergence between EBPs and usual practice. Transformational leaders inspire others and provide them a vision for what can be accomplished through extra personal effort and also encourage development and changes in mission that may lead therapists to have greater willingness to expend extra effort to learn new EBPs and push themselves to achieve more professionally [70]. Transformational leadership (individual consideration) was also associated with lower appeal and overall attitudes toward EBPs. One potential reason for this is that leaders who are more likely to focus on individual consideration may be more focused on the individual developmental needs of their therapists, some of which may be focused on more specific areas of development (e.g., diagnostic ability, common factors of psychosocial treatment that may promote recovery [77]) that may detract from a focus on EBPs.

### Associations between strategic organizational factors and knowledge and attitudes

Implementation climates that provided more educational support were associated with more positive attitudes toward EBPs, including higher ratings for being likely to adopt EBPs if they were required, higher ratings for appeal, lower levels of perceived divergence between EBPs and usual practice, and more positive overall attitudes toward EBPs. The educational support dimension of the ICS includes items about support for conferences, workshops, seminars, EBP trainings, training materials, and journals [26]. Enabling therapists to access these types of resources may give them opportunities to learn about EBPs and other effective practices and may also allow them to more clearly see the ways in which EBPs are similar to their preferred ways of working therapeutically. Implementation climates that were characterized by selecting staff who were more flexible, adaptable, and open to new interventions where associated with higher levels of the EBPAS subscale focusing on openness to EBPs. Implementation climates that fostered more financial or tangible rewards for EBPs were associated with less knowledge of EBPs and perceptions of higher levels of divergence between EBPs and routine practice. It is important to note that rewards for EBPs were rated extremely low in this sample of therapists and agencies (i.e., very limited range); however, it may be that providing financial reward for EBPs was more prevalent when knowledge of EBPs was low and that it could reinforce notions that they are highly divergent from therapists’ usual practices. It is also possible that these tangible rewards may undermine therapists’ intrinsic motivation to learn about EBPs [78].

Finally, more proactive implementation leadership was associated with greater appeal and more positive total EBPAS scores, and implementation leadership characterized as more knowledgeable was associated with less perceived divergence between EBPs and routine practice. Leaders that are proactive and have up-to-date knowledge of EBPs and are able to answer staff questions about them are likely to encourage therapists to view EBPs as a potentially feasible complement to their practice and dissuade them from thinking that EBPs are irrelevant or invalid treatment options. Perseverant implementation leadership was associated with lower willingness to adopt EBPs if required, less appeal of EBPs, and poorer overall attitudes toward EBPs. While it is difficult to interpret this finding, it may be that some leaders are more perseverant as an adaptive response to working in agencies comprised of therapists with poorer attitudes toward EBPs. It is also possible that the perseverance of leaders is being interpreted negatively by therapists, as they may perceive that leaders are being too dogmatic in attempting to implement EBPs in the face of all of the challenges associated with EBP implementation in large publicly funded behavioral health systems and that they may not be listening to or validating their concerns [15].

### Limitations

These findings should be viewed in light of several limitations. First, while we obtained a relatively high response rate (~50% of therapists employed at the 23 agencies), we did not have 100% participation and it is possible that nonparticipants’ views of their practice context differed from participants’ views. Second, the cross-sectional data preclude our ability to make causal statements about how the organizational context influences attitudes toward and knowledge of EBPs over time. Third, the organizations and therapists participating in this study were implementing a range of treatments, not all of which would be considered evidence-based. Variation in exposure to EBPs may have impacted their attitudes toward and knowledge of EBPs; however, the number of years the organizations participated in EBP initiatives was not predictive of either attitudes toward or knowledge of EBPs. Fourth, given the observational nature of this study and the inherent variation in the types of treatments implemented in community settings, we were unable to obtain objective ratings of fidelity. The variation in treatments implemented also precluded our ability to use innovation-specific versions of the ICS or the EBPAS, which may have greater predictive validity [24, 44].

### Future directions

This study suggests several important directions for the field. Improving our understanding of context will require continued efforts to develop and refine pragmatic measures of implementation determinants that would be relevant to behavioral health and health-care settings [29, 79]. Some of this work is underway. Lewis and colleagues [30] are working to develop criteria for pragmatic measures in implementation science, and there are an increasing number of measures in behavioral health and implementation research that are free, brief, and validated [25–27, 80, 81]. Efforts to document the predictive validity of measures are particularly needed [29, 30]. The mounting evidence of the impact of organizational-level factors on implementation outcomes suggests a need for interventions that more explicitly target the organizational context. Opportunities to develop, refine, and test organizational-level implementation strategies abound [82, 83], as a recent Cochrane review of interventions to improve organizational culture in health care returned no studies that met the inclusion criteria [84]. In behavioral health, interventions such as the Availability, Responsiveness, and Continuity (ARC) [42, 85, 86] and Leadership and Organizational Change (LOCI) [49] interventions demonstrate the utility of implementation strategies that target organizational factors, and serve as exemplars for the development of interventions that will allow organizations and systems to adaptively respond to the implementation challenges that emerge across different innovations and phases of implementation. These implementation strategies should address both factors associated with general organizational contexts (e.g., organizational culture, organizational climate, transformational leadership) and factors associated with strategic organizational contexts (e.g., implementation climate, implementation leadership), exploring their potential roles as mediators and moderators of implementation effectiveness [87]. The use of mixed methods will complement those efforts, adding nuance to our understanding of when and how contextual factors influence proximal and distal outcomes related to the implementation of effective practices (e.g., [88–90]). Further, system science methods might be leveraged to better understand the dynamic complexity of contextual determinants [91, 92].

## Conclusions

This study provides further evidence that both general and strategic organizational factors are important determinants to consider. While both general and strategic factors predicted knowledge of and attitudes toward EBPs, it is possible that the strategic organizational factors could be more feasible targets for change, as it may be somewhat easier to promote the improvement in implementation climate and leadership as compared to changing more durable general organizational determinants such as organizational culture and climate, which are expensive and time-consuming to address [43]. However, it is also possible that developing solid general organizational context and leadership capacity is a necessary precursor to the formation of effective implementation climate and leadership. We suggest that each of these organizational determinants may be relevant to a wide range of implementation studies and merit further research to determine how these constructs (and their associated subscales) influence implementation across a diverse array of interventions and settings.

## Abbreviations

ARC: Availability, Responsiveness, and Continuity
DBHIDS: Philadelphia’s Department of Behavioral Health and Intellectual disAbility Services
EPIC: Evidence-Based Practice and Innovations Center
EBPAS: Evidence-Based Practice Attitudes Scale
EBPs: Evidence-based practices
ICS: Implementation Climate Scale
ILS: Implementation Leadership Scale
KEBSQ: Knowledge of Evidence-Based Services Questionnaire
LOCI: Leadership and Organizational Change Intervention
OSC: Organizational Social Context Measurement System
